# Alzheimer's Disease cerebrospinal fluid Biomarkers and kidney function in normal and cognitively impaired older adults

**DOI:** 10.1002/alz.093374

**Published:** 2025-01-09

**Authors:** Reem M. Neal, zhiyi Yang, James J. Lah, Ihab Hajjar

**Affiliations:** ^1^ UT Southwestern Medical Center, Dallas, TX USA; ^2^ Emory University, Atlanta, GA USA

## Abstract

**Background:**

Recent Alzheimer’s disease (AD) clinical trials have used CSF biomarker levels for screening and enrollment. Preliminary evidence suggests Alzheimer’s Disease (AD) risk may be related to impaired renal function but the association of variation in levels of biomarkers commonly used AD biomarkers with kidney function is unknown.

**Method:**

We conducted an analysis using data from participants enrolled in two research protocols at the Goizueta Alzheimer’s Disease Research Center that had simultaneous measurements of serum creatinine at the time of their Cerebrospinal fluid (CSF) collection (N=970). The participants had a mean age of 66.5 years, with 52% showing mild cognitive impairment (MCI). Among them, 31% were of African American ethnicity, and 62% were women. Estimated glomerular filtration rate (eGFR) was obtained from chronic kidney disease‐epidemiology Collaboration. All participants had similar CSF collection procedures. Aβ42, Tau, or pTau were measured on the Luminex ALZBIO platform. General linear models and individual data were used to assess relationships between biomarkers and eGFR.

**Result:**

The analysis revealed significant associations between all three biomarkers and eGFR. The slopes per unit of eGFR were as follows: 0.75 (p=0.002) for Aβ42, ‐0.39 (p<0.0001) for Tau, and ‐0.13 (p=0.0002) for pTau. The association between eGFR and AD biomarkers was independent of cognitive status. However, there was a notable interaction between MCI and eGFR, where the impact of eGFR on AD biomarker levels was more robust in individuals with cognitive impairment (interaction p‐values: MCI*GFR: 0.005, Tau: 0.04, pTau: 0.04)

**Conclusion:**

We found a significant association between eGFR with CSF levels of Aβ42, tau, and pTau that were stronger in those with mild cognitive impairment. This suggests that kidney function should be considered in the context of interpreting the positivity of AD biomarkers in clinical evaluations and clinical trials, especially in those in the early symptomatic stage of AD. Future studies need to further explore the impact of kidney function and other comorbidities on the interpretation of AD Biomarkers.

